# Evaluation of Pediatric Early Warning Score (PEWS) in Unintentional Childhood Injuries Admitted to the Critical Care Unit

**DOI:** 10.7759/cureus.65312

**Published:** 2024-07-24

**Authors:** Sagar Malde, Prajvi Jain, Natesan Revathi, Bageshree Seth, Maninder S Setia

**Affiliations:** 1 Pediatrics, Mahatma Gandhi Missions (MGM) Medical College, Navi Mumbai, IND; 2 Epidemiology, Mahatma Gandhi Missions (MGM) Institute of Health Sciences, Navi Mumbai, IND

**Keywords:** positive predictive value (ppv), adverse clinical outcomes, pediatric early warning score (pews), epidemiology study, unintentional childhood injuries

## Abstract

Introduction: Pediatric Early Warning Score (PEWS), also known as Brighton PEWS or Monaghan PEWS, was developed to identify children at risk for clinical deterioration in hospitals. We designed this study to describe the epidemiology of unintentional injuries in children admitted to the critical care unit in a tertiary healthcare setting, and to determine the predictive properties of PEWS in these injuries.

Methods: This is a cross-sectional study. Injury-related data were based on Haddon’s matrix of agent, host, and environment factors. Each child was evaluated using PEWS on admission. We noted the following outcomes: duration of stay in the intensive unit; major intervention required; and death.

Results: We analyzed data from 157 children. Most of the children were in the age group of one to five years (57.7%), followed by more than five to 12 years old (37.6%). The most common injuries were bites (35.7%), falls (24.2%), and poisoning (21.7%). These injuries occurred at home (52.7%) and in the presence of a caretaker (40.0%). On admission, 11% of children were classified as green, 40% as yellow, 36% as orange, and 13% as red by PEWS. Classification of red versus the rest (orange/yellow/green) had a high sensitivity (100%), specificity (88.3%), and negative predictive value (100%) for “death” as an outcome.

Conclusion: Most of the injuries occurred at home and at a time when the caretaker was around. Thus, it will be useful to develop “safe home interventions” and train parents in first aid to take care of these injuries on-site. Baseline PEWS was a good predictor of “poor” as well as “positive” outcomes. It may be worthwhile to implement this score regularly in the management of childhood injuries in hospitals.

## Introduction

Globally, injuries account for nearly 8% of total deaths and are among the top five leading causes of death [1]. Unintentional and transport-related injuries were responsible for 25% of the deaths in adolescents and 14% of disability-adjusted life years (DALYs) globally; it was higher in males compared with females [2]. Injuries account for about 6% of total deaths in children under the age of five years in India, and a recent study reported that the prevalence of unintentional injuries in children was 4.7% (95% confidence intervals 4.4% to 4.9%); falls were the most common and drowning-related injuries were the least common injuries [3,4]. Furthermore, even though the prevalence of injuries was higher in male children, burn-related injuries and poisonings were more common in females [5]. Thus, there appear to be epidemiological differences according to age and gender [6]. Furthermore, childhood injuries may also increase mental health illness, substance abuse, and social issues in these children [1].

Thus, it is important to manage these critical injuries appropriately in healthcare settings. One of the components of management is the early assessment of children admitted to emergency units and intensive care units. Pediatric Early Warning Score (PEWS) also known as Brighton PEWS or Monaghan PEWS was developed to identify children at risk for clinical deterioration in hospitals [7,8]. It also helps to identify children who may require intensive care or consultation [9]. The use of PEWS has increased in hospital settings from 2005 onward [10]. However, some authors argue that even though there is an increase in the use of PEWS, there is limited evidence of its effectiveness [10]. Other authors have, however, found that PEWS had a good predictive value for identifying poor outcomes (respiratory or cardiac arrest or unexpected deaths) [11]. Another recent systematic review found that even though PEWS is useful in the improvement of clinical and process outcomes, there was limited evidence of its role as a healthcare intervention [12]. There are other scores such as the pediatric index of mortality (PIM), the pediatric risk of mortality score (PRISM), and the pediatric logistic organ dysfunction-2 (PELOD-2); these scores have been used as predictors of mortality in intensive care units [13-15]. These scores usually require calculators and include many more parameters compared with PEWS. PRISM uses 14 parameters, the PIM-2 score has 10 variables, and the PELOD-2 uses 10 variables from five organ dysfunctions; these are clinical and biochemical parameters [13,16,17]. PEWS, however, is easy to score using three parameters (behavior, cardiovascular, and respiratory), and its implementation is associated with a reduction in clinical deterioration and hospital mortality - even in resource-limited settings [7,18,19]. Hence, if proven to be useful, it may be easy to replicate and use in rural and resource-limited settings.

With this background, we designed the present study with these specific objectives: 1) to describe the epidemiological profile of unintentional injuries in children admitted to the critical care unit in teaching hospital located in resource-limited settings and 2) to evaluate the test properties (sensitivity, specificity, positive predictive value (PPV), negative predictive value (NPV), and area under the curve (AUC)) of PEWS for clinical outcomes of these injuries.

## Materials and methods

The present study is a cross-sectional analysis of data collected from children who presented with unintentional injuries and were admitted to the critical care unit over a period of 17 months from February 2017 to July 2018.

Study site and population

The study was conducted in the Pediatric Intensive Care Unit (PICU) of the Department of Pediatrics of MGM Hospital, Navi Mumbai, India. This is a teaching hospital located about 50 km from Mumbai. The hospital has an eight-bed PICU, with 45-50 admissions per month. A consecutive sample of children aged one month to twelve years of age who presented with unintentional injuries in the emergency department and were admitted to the PICU were included in the present analysis. The cases which were proven or suspected of intentional injuries were excluded.

Study procedure and variables

Detailed information was recorded in a pre-designed form. The variables in the data collection sheet were based on Haddon’s matrix [20] of host, environment, and agent factors. The host factors were age; sex; and socio-economic class based on the Kuppuswamy scale (divided into five groups - upper, upper middle, lower middle, upper lower, and lower) [21]. The environmental factors were time of injury (morning 8 am to 4 pm, evening 4 pm to 10 pm, night 10 pm to 8 am); day of injury (weekday and weekend); place of injury (road, home, school, playground, others); presence of caretaker (nobody, parent or other); and time to hospitalization (up to one hour, one hour to <6 hours, >=6 hours). At the time of presentation, the injuries were classified into the following types: accidental poisoning; foreign body injuries; drowning; bites; burns; falls; and road traffic accidents (RTAs). For analysis in the tables, we have grouped these into poisoning, bites, falls, RTAs, and others (due to small numbers in these categories). We classified age into three categories: up to 12 months of age (neonates and infants); more than 12 months to five years of age (toddlers and preschool children), and more than five years to 12 years of age (school-going children). The care of the child and movement within and outside the house may differ in these groups. We also recorded details on agent factors: poisoning (such as kerosene, insect repellant, rat poison, camphor, turpentine oil, diesel, gas cleaner, or any other agent), type of foreign body ingestion, drowning (such as fresh water, seawater, bucket, or bathtub), bites (cause of bites and the body part affected), burns (thermal or electric), fall (fall from height, fall from stairs, fall from the level ground; and the body parts injured), and road traffic accidents (type of injury - abrasion, contusion, laceration, or fracture, body part affected, and whether the injured child was the pedestrian or the passenger).

At the time of admission to the PICU, each child was evaluated using the PEWS [7,19]. Three parameters were considered for allocating scores: behavior; cardiovascular; and respiratory. They were scored on a scale of 0-3. The components included the following parameters: 1) behavior including patient’s level of consciousness (playing/appropriate, sleeping, irritable, and lethargic/reduced response to pain); 2) cardiovascular parameters such as color or capillary refill time and heart rate (based on age-appropriate cut-offs); and 3) respiratory parameters included respiratory rate (age-specific mean respiratory rates), use of accessory muscles, sternal recession, tracheal tug and grunting, liters of O2 needed for maintaining saturation of 98%-100%. An additional score of 2 was added for ¼ hourly nebulizers or persistent vomiting following surgery [7, 19]. PEWS were graded as Green (0-2), Yellow (3), Orange (4), or Red (5+) indicating low(green), medium (yellow or orange), and high (red) risk for deterioration. We also recorded the clinical course of the child in the PICU. We recorded the following outcomes in the PICU: duration of stay in the PICU, any major intervention (defined as ventilation or surgical procedure) required during the stay in the PICU, and death.

Statistical analysis 

With a sample size of 157, we had >90% power and alpha of 0.05, to detect an AUC of 0.86 from the receiver operating characteristics (ROC) curve for ICU admission for PEWS score (based on a previous study by Gold and colleagues [19]). This also included a 5% inflation of sample size to account for missing data. The sample size was estimated using the formula for “Sample Size for Single ROC curve.” This sample size also had enough power for epidemiological data of 35% of road traffic accidents (also reported previously [22]). The sample size of 157 had 90% power, with an alpha of 0.05 and a delta of 0.125. The sample size was estimated using Stata version 17 (StataCorp, College Station, TX, USA).

Data were entered in MS Excel (Microsoft, Washington, DC, USA) and analyzed using Stata version 15.1 (Stata Corp.). We estimated the mean and standard deviation (SD) for the linear variable. We estimated the proportions for categorical variables; these proportions across various groups were compared using the chi-square test or Fisher’s exact test for low expected cell counts. The means were compared using the t-test for two groups or analysis of variance for more than two groups. We then used the logistic regression models for multivariate analysis and estimated the odds ratio (OR) and their 95% confidence intervals (CI). These models were used to study the factors (host and environment factors) associated with different types of injuries. We also estimated the diagnostic test properties of the PEWS for clinical outcomes (stay for more than one day, major intervention required, and death) in these children. We plotted the receiver operating characteristics curve (ROC). We estimated the AUC from these ROC curves, sensitivity, specificity, PPV, and NPV for these diagnostic test properties. A “p” value of <0.05 was considered statistically significant. The study was approved by the Ethics Committee at Mahatma Gandhi Missions (MGM) Medical College, Navi Mumbai, India (Reference No. 2017/04/SC/76, date: May 2, 2017).

## Results

We present the results from 157 children admitted to the intensive care unit. The majority of the children admitted with unintentional injuries to the critical care unit were in the age group of one to five years (57.7% (n=91)), followed by more than five years to 12 years (37.6% (n=59)); the proportion of males was higher (58.6% (n=92)) compared with females (41.4% (n=65)). The children were in the lower (26.1% (n=41)) and upper lower (69.4% (n=109)) socio-economic status. The most common injuries were bites (35.7% (n=56)), falls (24.2% (n=38)), and poisoning (21.7% (n=34)). The majority of the injuries occurred in the evenings (53.5% (n=84)) and weekdays (73.9% (n=116)). Most of the injuries were at home (52.7% (n=83)), on the playground (19.7% (n=31)), and on roads (17.9% (n=28)). Though a caretaker (mother) was present at the time of injury in about 40% (n=62) of the cases, no caretaker was present in 26% (n=41) of these cases. We have presented detailed descriptive data in Table 1.

**Table 1:** Number and proportions (%) according to host and environment factors in 157 children with injuries admitted to the critical care unit, Navi Mumbai, India The total column uses 157 as the denominator and the other % are for row total except for total (which is column percentage)

Characteristics	Total	Poisoning	Bites	Falls	Road traffic accidents	Others	P-value
	N (%)	n (%)	n (%)	n (%)	n (%)	n (%)	
All children	157 (100)	34 (21.7)	56 (35.7)	38 (24.2)	19 (12.1)	10 (6.4)	
Host factors							
1 month - 12 months	7 (4.5)	1 (14.3)	3 (42.9)	3 (42.9)	0 (0)	0 (0)	<0.001
>12 months - 5 years	91 (57.7)	28 (30.8)	18 (19.8)	25 (27.5)	11 (12.1)	9 (9.9)	
>5 years - 12 years	59 (37.6)	5 (8.5)	35 (59.3)	10 (16.9)	8 (13.6)	1 (1.7)	
Sex							
Female	65 (41.4)	18 (27.7)	18 (27.7)	14 (21.5)	9 (13.8)	6 (9.2)	0.21
Male	92 (58.6)	16 (17.4)	38 (41.3)	24 (26.1)	10 (10.9)	4 (4.4)	
Socio-economic class							
Lower middle	7 (4.5)	4 (57.1)	1 (14.3)	1 (14.3)	0 (0)	1 (14.3)	0.001
Upper lower	109 (69.4)	18 (16.5)	32 (29.4)	34 (31.2)	17 (15.6)	8 (7.3)	
Lower	41 (26.1)	12 (29.3)	23 (56.1)	3 (7.3)	2 (4.9)	4 (9.1)	
Environment factors							
Time of injury							
Morning	59 (37.6)	18 (30.5)	15 (25.4)	19 (32.2)	3 (5.1)	4 (6.8)	0.031
Evening	84 (53.5)	14 (16.7)	32 (38.1)	18 (21.4)	14 (16.7)	6 (7.1)	
Night	14 (9.0)	2 (14.3)	9 (64.3)	1 (7.1)	2 (14.3)	0 (0)	
Day of week							
Weekday	116 (73.9)	27 (23.3)	40 (34.5)	27 (23.3)	16 (13.8)	6 (5.2)	0.58
Weekend	41 (26.1)	7 (17.1)	16 (39.0)	11 (26.8)	3 (7.3)	4 (9.8)	
Time to hospitalization							
Up to 1 hour	92 (58.6)	25 (27.2)	18 (19.6)	30 (32.6)	12 (13)	7 (7.6)	<0.001
>1 hour to <6 hours	62 (39.5)	8 (12.9)	37 (59.7)	8 (12.9)	6 (9.7)	3 (4.8)	
>=6 hours	3 (1.9)	1 (33.3)	1 (33.3)	0 (0)	1 (33.3)	0 (0.0)	
Place of injury							
Road	28 (17.9)	0 (0)	7 (25.0)	2 (7.1)	19 (67.9)	0 (0)	<0.001
Home	83 (52.7)	30 (36.1)	21 (25.3)	26 (31.3)	0 (0)	6 (7.2)	
School	5 (3.2)	0 (0)	1 (20.0)	3 (60.0)	0 (0)	1 (20.0)	
Playground	31 (19.7)	4 (12.9)	20 (64.5)	6 (19.4)	0 (0)	1 (3.2)	
Others	10 (6.4)	0 (0)	7 (70.0)	1 (10.0)	0 (0)	2 (20.0)	
Caretaker at time of injury							
Father	18 (11.5)	2 (11.1)	10 (55.6)	1 (5.6)	5 (27.8)	0 (0)	<0.001
Mother	62 (39.5)	21 (33.9)	9 (14.5)	18 (29.0)	9 (14.5)	5 (8.1)	
Both parents	24 (15.3)	3 (12.5)	4 (16.7)	12 (50.0)	4 (16.7)	1 (4.2)	
Relatives	0 (0)	0 (0)	0 (0)	0 (0)	0 (0)	0 (0)	
Neighbor	2 (1.3)	0 (0)	2 (100.0)	0 (0)	0 (0)	0 (0)	
Others	10 (6.4)	0 (0)	5 (50.0)	3 (30.0)	0 (0)	2 (20.0)	
None	41 (26.1)	8 (19.5)	26 (63.4)	4 (9.8)	1 (2.4)	2 (4.9)	

In the multivariate analysis, we found that poisoning was significantly more common at home than in other places (OR: 8.34, 95% CI: 2.46, 28.29; p < 0.001). Bites were less likely in children aged one to five years compared with those up to 12 months (OR: 0.13, 95% CI: 0.02, 0.94; p < 0.05) (reference category up to 12 months). Bites were also significantly less likely when parents were present (OR: 0.19, 95% CI: 0.07, 0.48; p < 0.01); however, they were significantly more likely in the night (OR: 10.42, 95% CI: 2.23, 48.71; p < 0.05). Falls were significantly less likely at night compared with morning (OR: 0.11, 95% CI: 0.01, 0.99; p < 0.05) (reference category - “morning”). Road traffic accidents were significantly more common when children were with parents compared with when parents were not present (OR: 12.51, OR: 1.53, >100.0; p < 0.01) (reference category - “parents not present”). We have presented ORs and their 95% confidence intervals for these multivariate models in Table 2.

**Table 2:** Odds ratios (ORs) and 95% confidence intervals (CIs) of association between host and environment factors and the type of injury Significance *p<0.05, **p<0.01

Variables	Poisoning	Bites	Falls	Road traffic accidents
	OR (95% CIs)	OR (95% CIs)	OR (95% CIs)	OR (95% CIs)
Age group				
1 month - 12 months	Reference	Reference	Reference	Reference
>12 months - 5 years	6.16 (0.63, 59.92)	0.13 (0.02, 0.94)*	0.56 (0.09, 3.40)	Reference
>5 years - 12 years	2.85 (0.24, 33.86)	0.72 (0.10, 5.42)	0.38 (0.05, 2.67)	1.57 (0.52, 4.76)
Sex				
Female	Reference	Reference	Reference	Reference
Male	0.48 (0.20, 1.18)	2.25 (0.94, 5.37)	1.57 (0.68, 3.63)	0.71 (0.25, 2.01)
Socioeconomic class				
Lower middle	Reference	Reference	Reference	Reference
Upper Lower	0.32 (0.05, 1.90)	1.29 (0.11, 14.64)	6.22 (0.66, 58.26)	Reference
Lower	0.67 (0.10, 4.40)	5.86 (0.48, 70.88)	0.95 (0.08, 11.34)	0.35 (0.07, 1.74)
Caretaker				
No parents	Reference	Reference	Reference	Reference
Parents present	1.07 (0.35, 3.27)	0.19 (0.07, 0.48)**	1.64 (0.59, 4.56)	12.51 (1.53, >100)*
Time of day				
Morning	Reference	Reference	Reference	Reference
Evening	0.64 (0.25, 1.67)	1.98 (0.76, 5.18)	0.61 (0.26, 1.44)	3.77 (0.98, 14.67)
Night	--	10.42 (2.23, 48.71)*	0.11 (0.01, 0.99)*	2.10 (0.28, 15.67)
Day of week				
Weekday	Reference	Reference	Reference	Reference
Weekend	0.52 (0.17, 1.54)	1.04 (0.39, 2.79)	1.40 (0.56, 3.53)	0.47 (0.12, 1.89)
Place of injury				
Not home	Reference	Reference	Reference	--
At home	8.34 (2.46, 28.29)**	0.88 (0.33, 2.32)	2.02 (0.80, 5.09)	--

The mean (SD) PEWS on admission was 3.48 (1.23). The mean (SD) PEWS was significantly higher in children who died compared with those who did not (6.33 (1.15) vs 3.42 (1.18); p<0.001). Similarly, the mean (SD) PEWS values were significantly higher in those who had any “major intervention” compared with those who did not (5.33 (1.53) vs 3.44 (1.21); p=0.01). We have presented the means and SDs in Table 3. On admission, 11% (n=17) of children were classified as green, 40% (n=62) as yellow, 36% (n=57) as orange, and 13% (n=21) as red by PEWS. There was no association between age and category of PEWS (p=0.08). There was a significant association between the type of injury and PEWS scoring (p<0.001). A higher proportion of burns and road traffic accidents were classified as orange or red on the PEWS score (Table 4). Among those who were admitted to the PICU for only one day (n=68), 4% (n=3) were classified as red on admission; the proportion was 15% (n=8) in those who were admitted to the PICU for two days (n=53), and it was 28% (n=10) in those who were admitted to the PICU for more than two days (n=36). There appeared to be a significant association between the duration of stay in the PICU and PEWS classification on admission (p < 0.01). We also found that all the deaths were classified as red on admission. All children who required major intervention were classified as orange or red on PEWS. We have presented detailed proportions for PEWS categories in Table 4.

**Table 3:** Means and standard deviations (SDs) of Pediatric Early Warning System (PEWS) score according to demographic and injury-related characteristics, Navi Mumbai, India The total row shows column percentage, and the PEWS value column shows mean and standard deviation in parentheses

Characteristics	All	PEWS value	P-value
	N (%)	Mean (SD)	
Total	157 (100.0)	3.48 (1.24)	
Age			
1 month - 12 months	7 (4.5)	3.71 (1.11)	0.21
>12 months - 5 years	91 (58.0)	3.33 (1.07)	
>5 years - 12 years	59 (37.6)	3.68 (1.47)	
Type of injury			
Poisoning	34 (21.7)	3.29 (0.68)	0.04
Bites	56 (35.7)	3.75 (1.70)	
Falls	38 (24.2)	3.05 (0.73)	
Road traffic accidents	19 (12.1)	3.89 (0.99)	
Others	10 (6.4)	3.40 (1.17)	
Time to hospitalization			
< 1 hour	92 (58.6)	3.30 (0.90)	0.01
> 1 hour to < 6 hours	62 (39.5)	3.79 (1.54)	
>= 6 hours	3 (1.9)	2.33 (2.08)	
Outcome			
Discharge	0 (0)	--	<0.001
Transfer to ward	154 (98.1)	3.42 (1.18)	
Death	3 (1.9)	6.33 (1.15)	
Duration of stay in PICU			
1 day	68 (43.3)	2.71 (1.23)	<0.001
2 days	53 (33.8)	3.96 (0.88)	
> 2 days	36 (22.9)	4.22 (0.83)	
Major intervention required			
No	154 (98.1)	3.44 (1.21)	0.01
Yes	3 (1.9)	5.33 (1.53)	

**Table 4:** Number and proportions (%) of distribution of Pediatric Early Warning System (PEWS) score according to demographic and injury-related characteristics, Navi Mumbai, India The total column uses 157 as the denominator and the other %s are for row total except for total (which is column percentage) (significance * p<0.001)

Characteristics	All	PEWS-Green	PEWS-Yellow	PEWS-Orange	PEWS-Red
	N (%)	n (%)	n (%)	n (%)	n (%)
Total	157 (100.0)	17 (10.8)	62 (39.5)	57 (36.3)	21 (13.4)
Age					
1 month - 12 months	7 (4.5)	0 (0)	4 (57.1)	2 (28.6)	1 (14.3)
>12 months - 5 years	91 (58.0)	12 (13.2)	36 (39.6)	37 (40.7)	6 (6.6)
>5 years - 12 years	59 (37.6)	5 (8.5)	22 (37.3)	18 (30.5)	14 (23.7)
Type of injury*					
Poisoning	34 (21.7)	2 (5.9)	19 (55.9)	13 (38.2)	0 (0)
Bites	56 (35.7)	8 (14.3)	10 (17.9)	21 (37.5)	17 (30.4)
Falls	38 (24.2)	5 (13.2)	24 (63.2)	9 (23.7)	0 (0)
Road traffic accidents	19 (12.1)	0 (0)	7 (36.8)	9 (47.4)	3 (15.8)
Others	10 (6.4)	2 (20.0)	2 (20.0)	5 (50.0)	1 (10.0)
Time to hospitalization*					
Up to 1 hour	92 (58.6)	9 (9.8)	44 (47.8)	36 (39.1)	3 (3.3)
> 1 hour to < 6 hours	62 (39.5)	7 (11.3)	17 (27.4)	20 (32.3)	18 (29)
>= 6 hours	3 (1.9)	1 (33.3)	1 (33.3)	1 (33.3)	0 (0)
Outcome*					
Discharge	0 (0)	0 (0)	0 (0)	0 (0)	0 (0)
Transfer to ward	154 (98.1)	17 (11.0)	62 (40.3)	57 (37.0)	18 (11.7)
Death	3 (1.9)	0 (0)	0 (0)	0 (0)	3 (100.0)
Duration of stay in PICU*					
1 day	68 (43.3)	17 (25)	42 (61.8)	6 (8.8)	3 (4.4)
2 days	53 (33.8)	0 (0)	15 (28.3)	30 (56.6)	8 (15.1)
>2 days	36 (22.9)	0 (0)	5 (13.9)	21 (58.3)	10 (27.8)
Major Intervention required					
No	154 (98.1)	17 (11.0)	62 (40.3)	56 (36.4)	19 (12.3)
Yes	3 (1.9)	0 (0)	0 (0)	1 (33.3)	2 (66.6)

**Figure 1:**
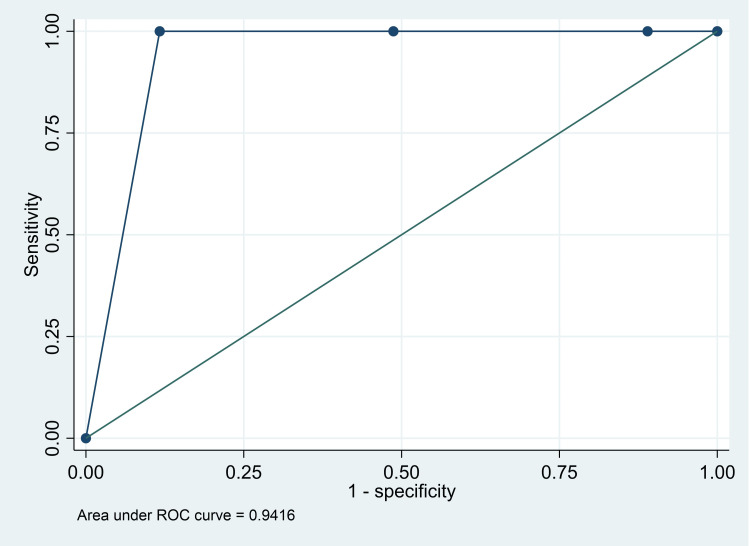
Receiver operating characteristics curve for PEWS score and “death” as an outcome PEWS: Pediatric Early Warning Score

**Figure 2:**
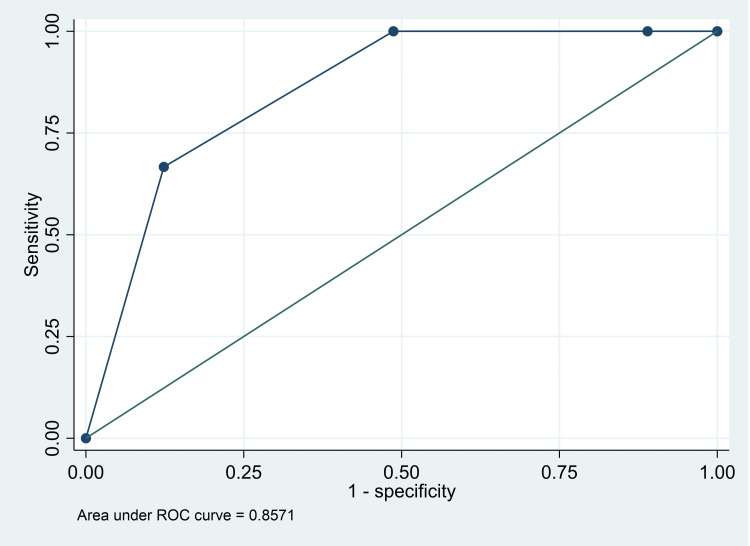
Receiver operating characteristics curve for PEWS score and “major intervention” as an outcome PEWS: Pediatric Early Warning Score

**Figure 3:**
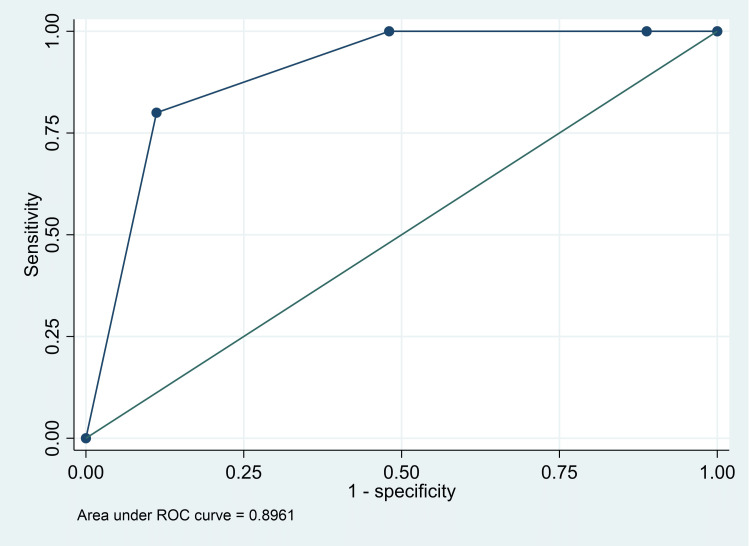
Receiver operating characteristics curve for PEWS score and “death or major intervention” as an outcome PEWS: Pediatric Early Warning Score

The AUC for death was 0.94 (95% CI: 0.92, 0.97), for major intervention was 0.86 (95% CI: 0.69, 1.00), and for either death or major intervention was 0.90 (95% CI: 80, 0.99) (Figures 1-3). The Classification of red versus the rest (orange/yellow/green) had a high sensitivity (100%; 95% CI: 29.2%, 100%), specificity (88.3%; 95% CI: 82.2%, 92.9%), and NPV (100%; 95% CI: 97.3%, 100%) for “death” as an outcome. However, the classification of red and orange versus yellow and green had good sensitivity (100%; 95% CI: 29.2%, 100%) and NPV (100%, 95% CI: 95.4%, 100%) for “major intervention required” as an outcome. Finally, classification of yellow and green versus orange and red had good sensitivity (86.8%; 95% CI: 76.4%, 93.8%), specificity (77.5%; 95% CI: 67.4%, 85.7%), PPV (74.7%; 95% CI: 63.6%, 83.8%), and NPV (88.5%; 95% CI: 79.2%, 94.6%) for “admission in the PICU for only one day” - considered as a good outcome (Table 5).

**Table 5:** Sensitivity, specificity, positive predictive value, negative predictive value, area under the curve (AUC) and their 95% confidence intervals (95% CI) of Pediatric Early Warning Score (PEWS) score in select clinical outcomes in children who presented with critical injuries, Navi Mumbai, India The data are presented as the estimate of measure along with its 95% confidence interval

Outcomes	Sensitivity (95% CI)	Specificity (95% CI)	Positive predictive value (95% CI)	Negative predictive value (95% CI)	AUC (95% CI)
Death					
Orange & red/yellow & green	100% (29.2%, 100%)	51.3% (43.1%, 59.4%)	3.9% (0.8%, 10.8%)	100% (95.4%, 100%)	0.76 (0.72, 0.80)
Red/orange & yellow & green	100% (29.2%, 100%)	88.3% (82.2%, 92.9%)	14.3% (3.1%, 36.3%)	100% (97.3%, 100%)	0.94 (0.92, 0.97)
Major intervention required					
Orange & red/yellow & green	100% (29.2%, 100%)	51.3% (43.1%, 59.4%)	3.9% (0.8%, 10.8%)	100% (95.4%, 100%)	0.76 (0.72, 0.80)
Red/orange & yellow & green	66.7% (9.4%, 99.2%)	87.7% (81.4%, 92.4%)	9.5% (1.2%, 30.4%)	99.3% (96.0%, 100%)	0.77 (0.44, 1.00)
Admitted for only 1 day in PICU					
Yellow & green/orange & red	86.8% (76.4%, 93.8%)	77.5% (67.4%, 85.7%)	74.7% (63.6%, 83.8%)	88.5% (79.2%, 94.6%)	0.82 (0.76, 0.88)
Green/yellow & orange & red	25% (15.3%, 37%)	100% (95.9%, 100%)	100% (80.5%, 100%)	73.6%, (55%, 71.5%)	0.63 (0.57, 0.68)

## Discussion

Thus, we found that the majority of the children admitted with critical injuries to the intensive care unit were in the age group of one to five years. The most common injuries were bites, falls, and poisoning (21.7%). In our study nearly half the children with critical injuries were classified as orange or red, and the other half as green or yellow. PEWS classification “red” had good sensitivity and specificity for “death” as an outcome; whereas PEWS classification “orange/red” had good sensitivity and specificity for “major intervention required.” However, the yellow/green classification on PEWS had good sensitivity for better outcomes - admission in the PICU for only one day.

The Global Burden of Disease dataset reported that unintentional injuries were responsible for about 686.49 DALYs per 100,000 population in children globally and 862.27 per 100,000 in India; it is the third cause of DALYs in India [23]. The important contributors to DALYs globally were road injuries (3.97%), drowning (2.50%), falls (1.31%), animal bites (0.82%), and foreign bodies (0.44%). In India, drowning (2.8%), road injuries (2.26%), animal bites (2.04%), and falls (1.5%) were the four important contributors to total DALYs [22]. In our study, bites and falls were the common injuries admitted to the PICU. Another study from Nigeria found that road traffic accidents, foreign bodies, and injuries were common childhood injuries [24]. However, another study from North India reported that falls (35%) were the most common childhood injuries [22]. We may have seen a lot of bites and falls compared with other Indian and international studies because the area covered by our hospital is largely rural and tribal with a hilly terrain. Thus, children are more likely to be exposed to animals (including wild animals), and due to the terrain, they are more likely to experience falls.

As seen in our study, the proportion of males presenting with injuries is higher compared with females [6,23]. A study from South India reported that the occurrence of road traffic accidents was nearly the same in males and females [25]; we found that the proportion was slightly higher in females compared with males. We had indicated earlier that the terrain covered by our hospital was hilly and tribal. Thus, girls who help their mothers in household work may be exposed to injuries as they try to negotiate the roads in this terrain. As seen in our population, most of the childhood injuries occurred at home [3]. Thus, it is important to create intervention programs aimed at increasing the safety of children at home (such as child-safe kitchens and water-holding areas) and train parents (particularly mothers) in first aid.

PEWS is an important early warning system in critical situations in children and is useful in identifying children at risk of deterioration in the ICU and monitoring the severity of clinical conditions [26,27]. In our study population - unintentional injuries admitted to the critical care unit - we did find that PEWS has good test properties not only in identifying children who may have poor outcomes but also those children who may have good outcomes (stayed in the ICU for one day only). The AUC was best for death followed by major intervention required in these children with injuries. Authors have also found that regular use of PEWS helped in improving interdisciplinary dynamics and emphasized the role of patient safety in healthcare settings [28]. Thus, it will be useful to include this bedside score in the triage of pediatric patients in the emergency unit, as well as in clinical management and decision-making for pediatric patients admitted to the hospital intensive care units or wards.

The study had its limitations. For instance, we monitored PEWS only at baseline (at the time of admission) to the intensive care unit. Though we did find baseline PEWS has good NPV for poor clinical outcomes in these children, repeated PEWS would probably be useful to monitor the progress of the child during the hospital stay. In addition, we did not include the facilitators and barriers to the administration of PEWS in clinical settings. Agulnik and colleagues have reported that beneficial outcomes in patients and support by supervisors/leaders were facilitators for its implementation, whereas staff turnover and COVID-19 hampered the regular administration of PEWS in hospital settings [29,30].

## Conclusions

Nonetheless, the study provides useful evidence on unintentional childhood injuries admitted to the critical care units and the role of PEWS for clinical outcomes of injuries in resource-limited healthcare settings. The most common injuries were bites, falls, and poisonings. Most of these injuries occurred at home and at a time when the caretaker (usually one or both parents) was around. Thus, we should develop “safe home interventions” to prevent injuries at home and parents should be trained in first aid to take care of these injuries on-site. Baseline PEWS had good NPV for “poor” and good PPV for “positive” outcomes in children who present with unintentional injuries. Easily administered, it may be worthwhile to implement PEWS regularly in the management of childhood injuries in hospitals in rural and resource-limited settings. Children who score green or yellow on the PEWS score may be managed in high-dependency units, and not overburden the intensive care units in resource-limited settings.
